# Supplementary figures and images for: Selective observation following betrayal shapes the social inference landscape
Source: PLoS Comput Biol. 2026 Apr 24;22(4):e1014200. doi: 10.1371/journal.pcbi.1014200 (PMC13193612; doi:10.1371/journal.pcbi.1014200)

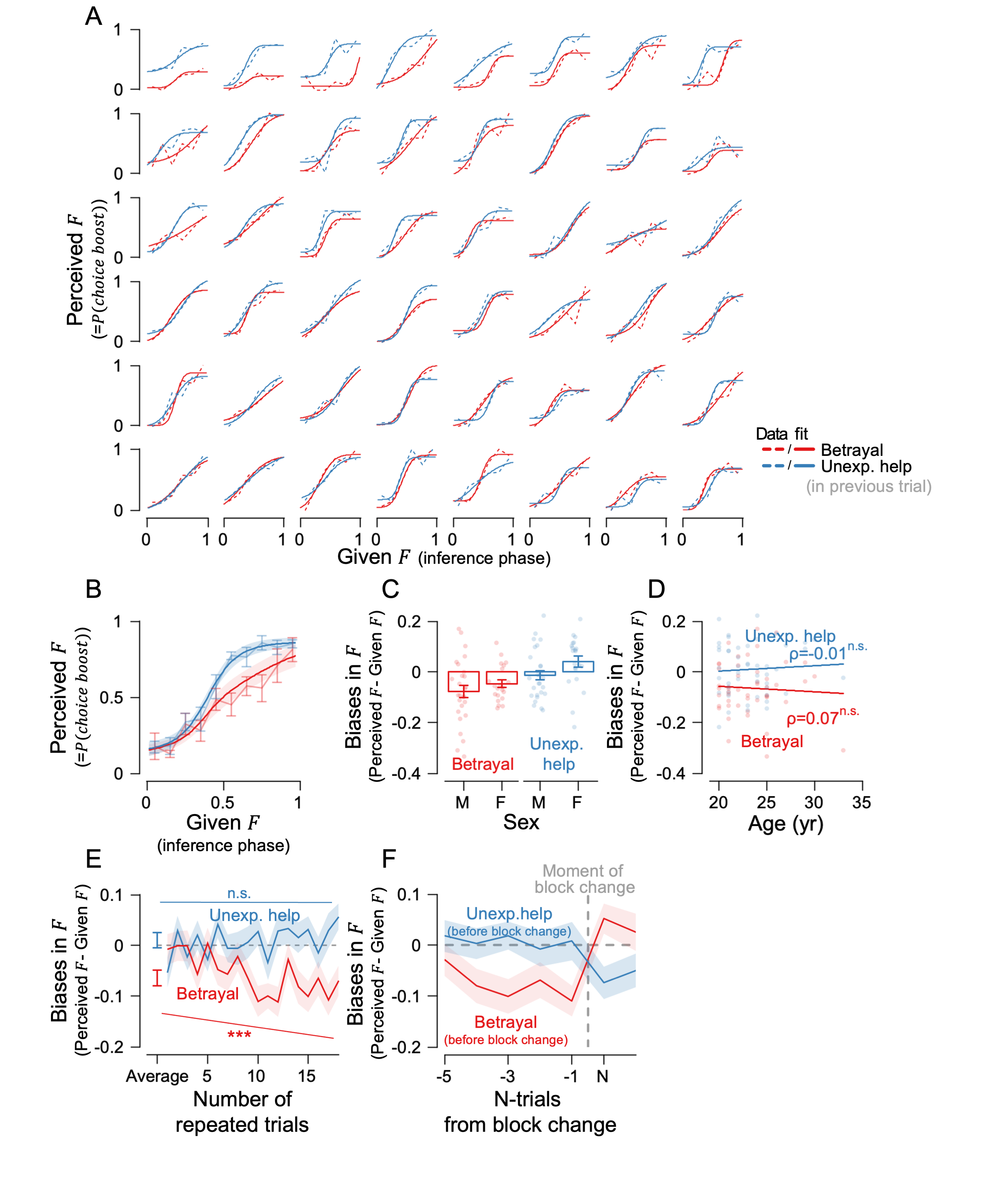

Supplement: S1 Fig — (A) The psychometric curves of F values for the forty participants. The solid line represents the cumulative Gaussian fit, while the dashed lines indicate the data. (B) Replication of the main effect in eight participants recruited in a separate session, showing the same pattern of results. (C) Biases in the inference are grouped by sex. (D) No correlation was found between participants’ age and biases in the inference. (E) Negative biases in perceived F value increased with repeated betrayal but did not after unexpected help. Stars indicate statistical significance (***, p < 0.001; n.s., not significant). (F) Biases in perceived F value re-aligned at the moment of block change. Colors indicate the block type before the block change occurred. Error bars and shaded ribbons represent ±1 SEM, and dots indicate individual participants. (TIFF) [file pcbi.1014200.s001.tiff]

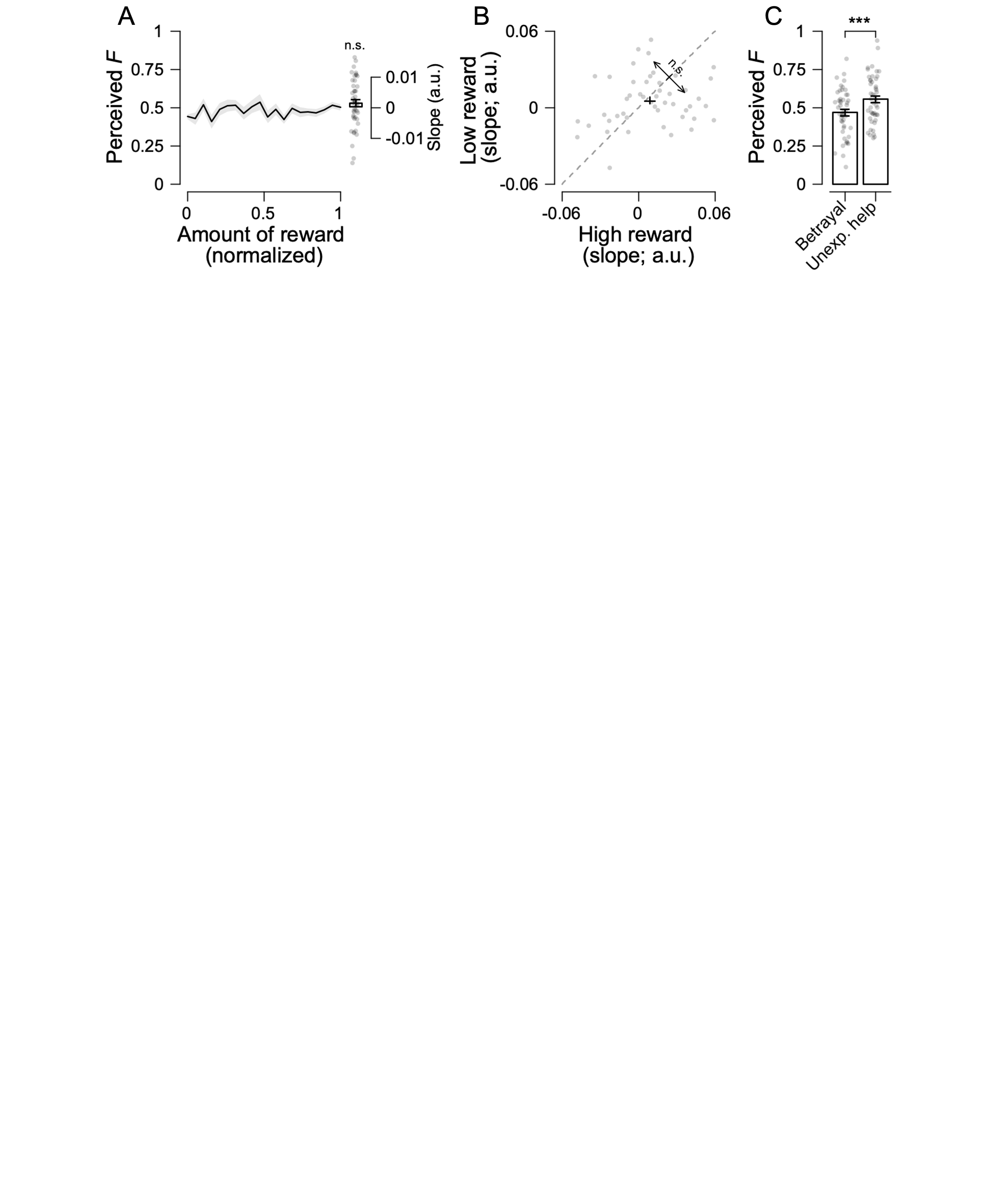

Supplement: S2 Fig — (A) Perceived F (i.e., the probability of choosing the boost item on the subsequent trial) as a function of normalized reward magnitude on the previous trial. The slope reflects each participant’s sensitivity to the reward from the previous trial. (B) Relationship between the slopes for low-reward and high-reward trials across participants. (C) Mean perceived F following betrayal and unexpected-help conditions within the just-noticeable-difference range. Error bars and shaded regions represent ±1 SEM; dots indicate individual participants. (TIFF) [file pcbi.1014200.s002.tiff]

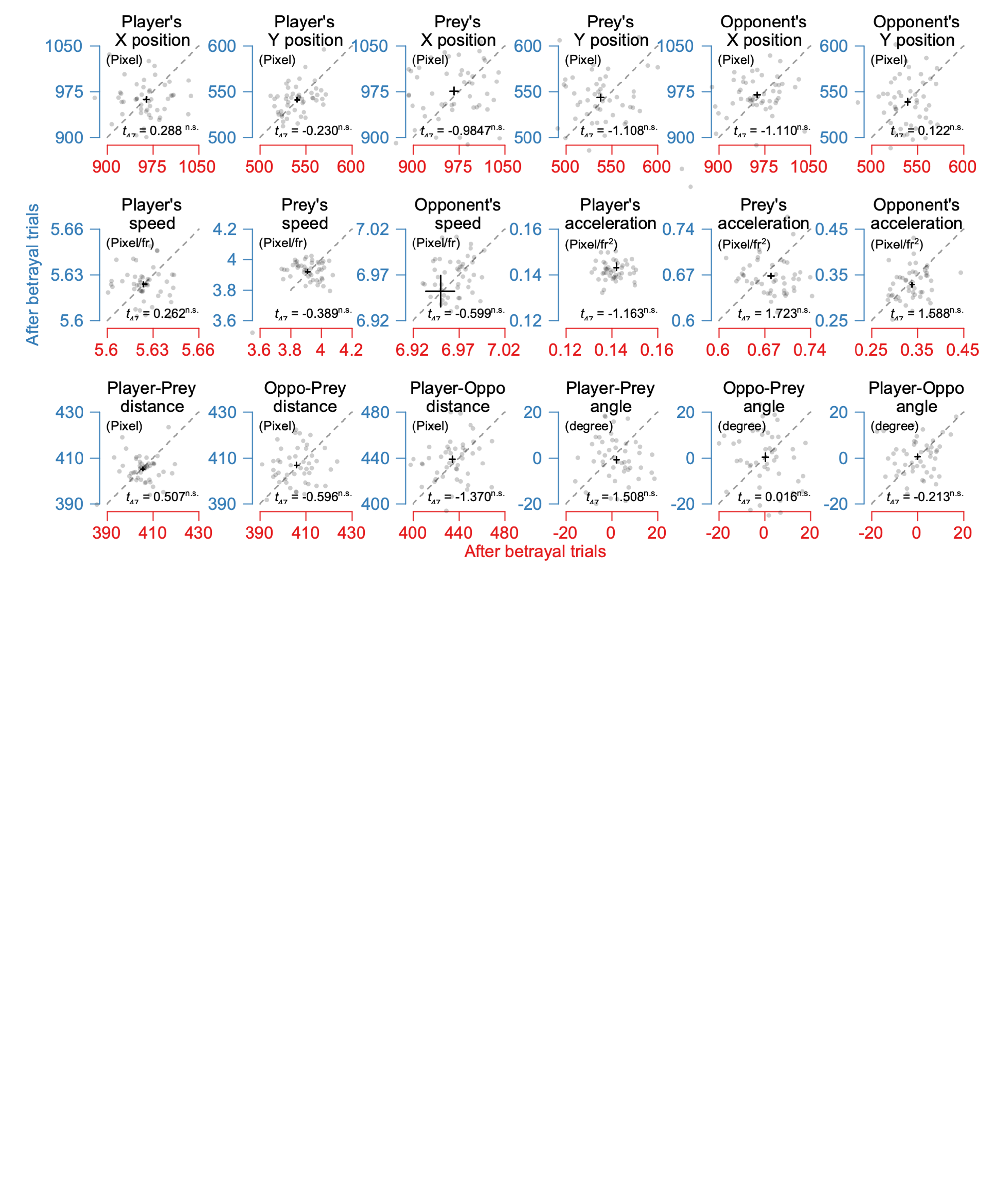

Supplement: S3 Fig — The position, speed, and acceleration of the player, prey, and opponent, as well as pairwise distances and movement angles among the three agents, were compared between trials following betrayal and trials following unexpected help. Crosses indicate mean ±1 SEM. ‘fr’ indicates frames (60 Hz sampling). (TIFF) [file pcbi.1014200.s003.tiff]

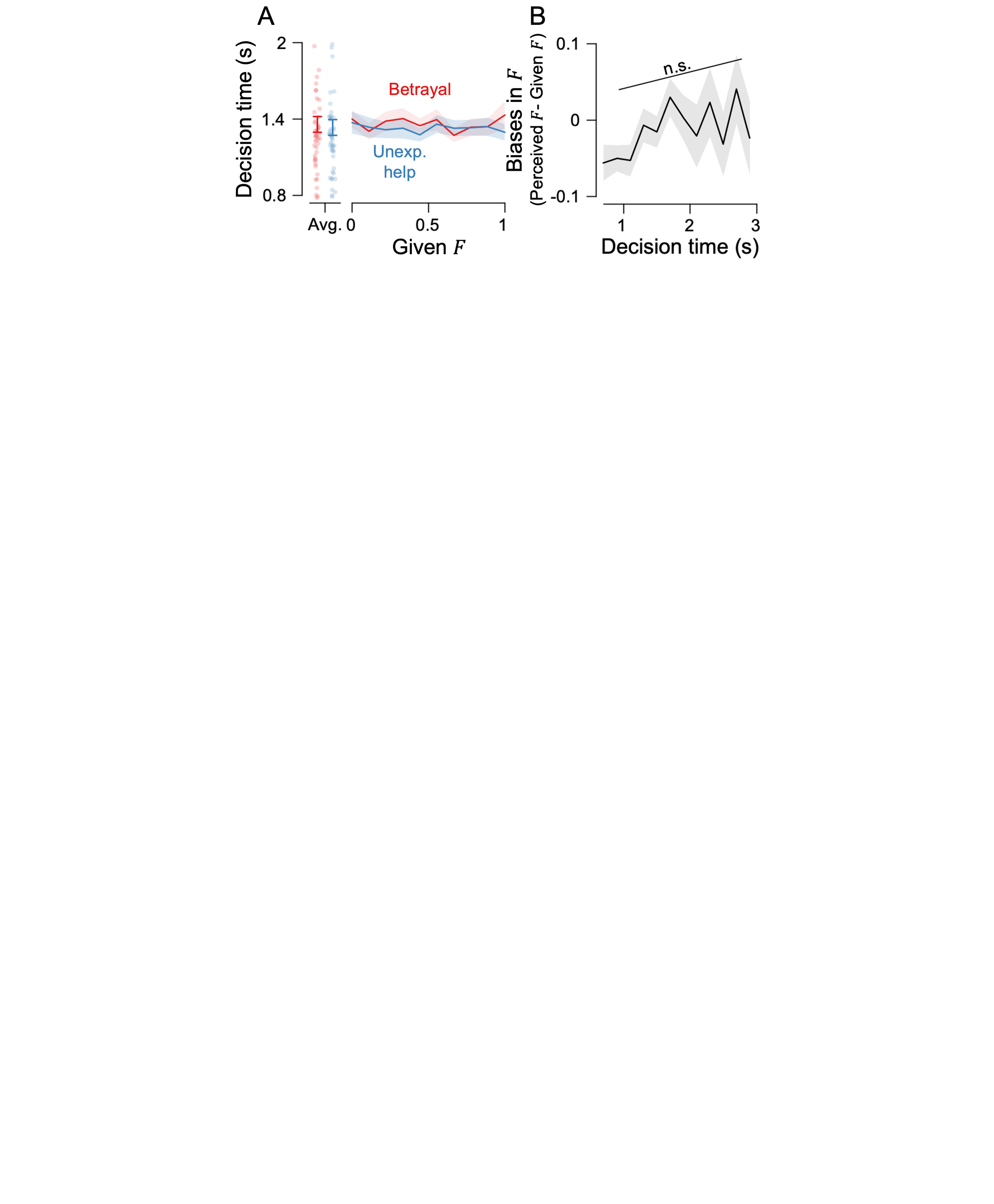

Supplement: S4 Fig — (A) No difference in decision times during the item decision phase between betrayal and unexpected help conditions. (B) Bias in perceived F as a function of decision time. Error bars and shaded ribbons represent ±1 SEM, and dots indicate individual participants. (TIFF) [file pcbi.1014200.s004.tiff]

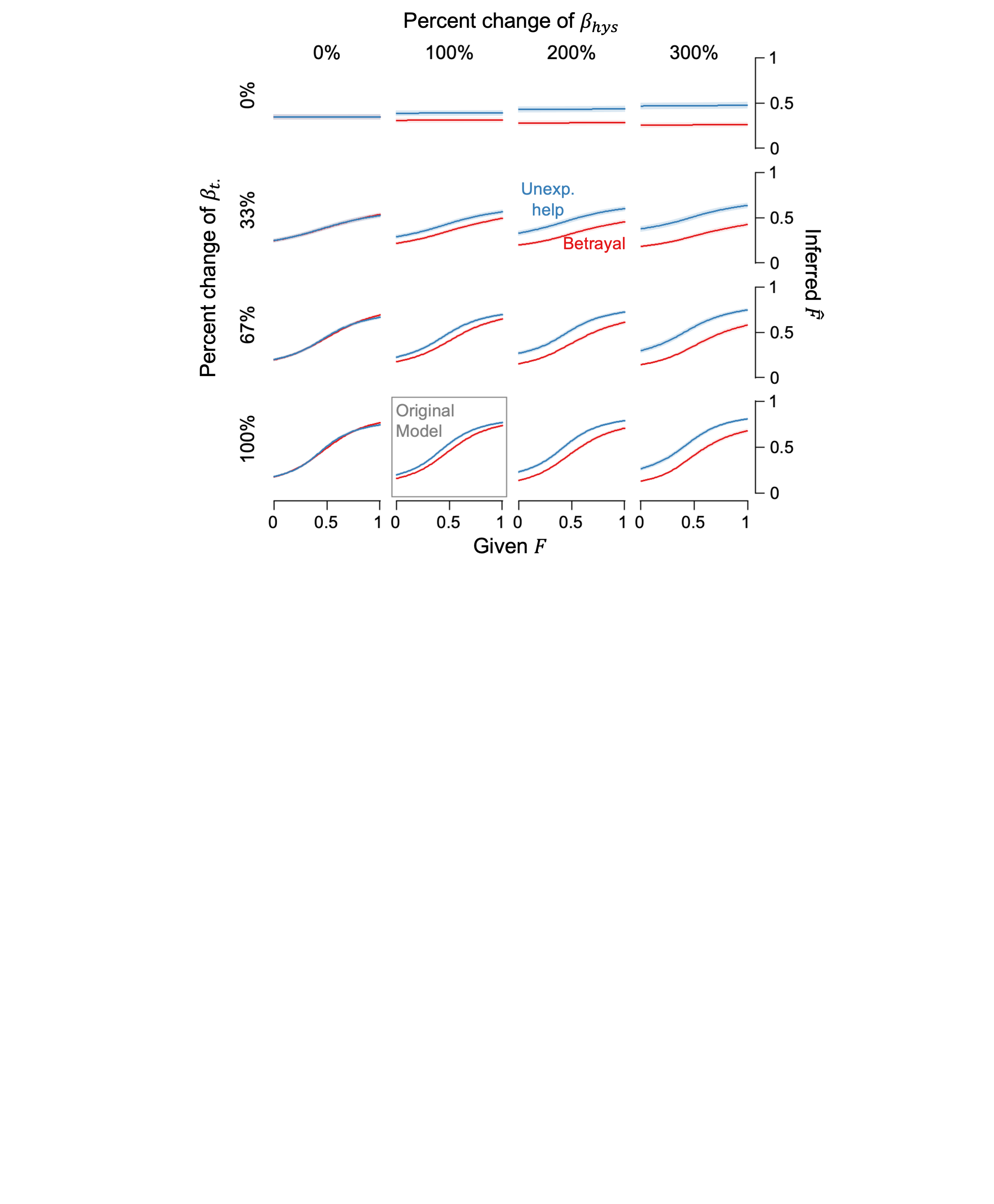

Supplement: S5 Fig — The weight on the experience of betrayal or unexpected help (βhys) and the weight on the three avatars’ trajectories (βt) were parametrically amplified or attenuated. Psychometric curves based on logistic regression model data capture inference during the last second before the item decision phase. Changes in βhys influenced the overall baseline difference between the betrayal and unexpected help conditions, while changes in βt affected the inferential bias in a given context. Shaded error bars represent ±1 SEM. (TIFF) [file pcbi.1014200.s005.tiff]

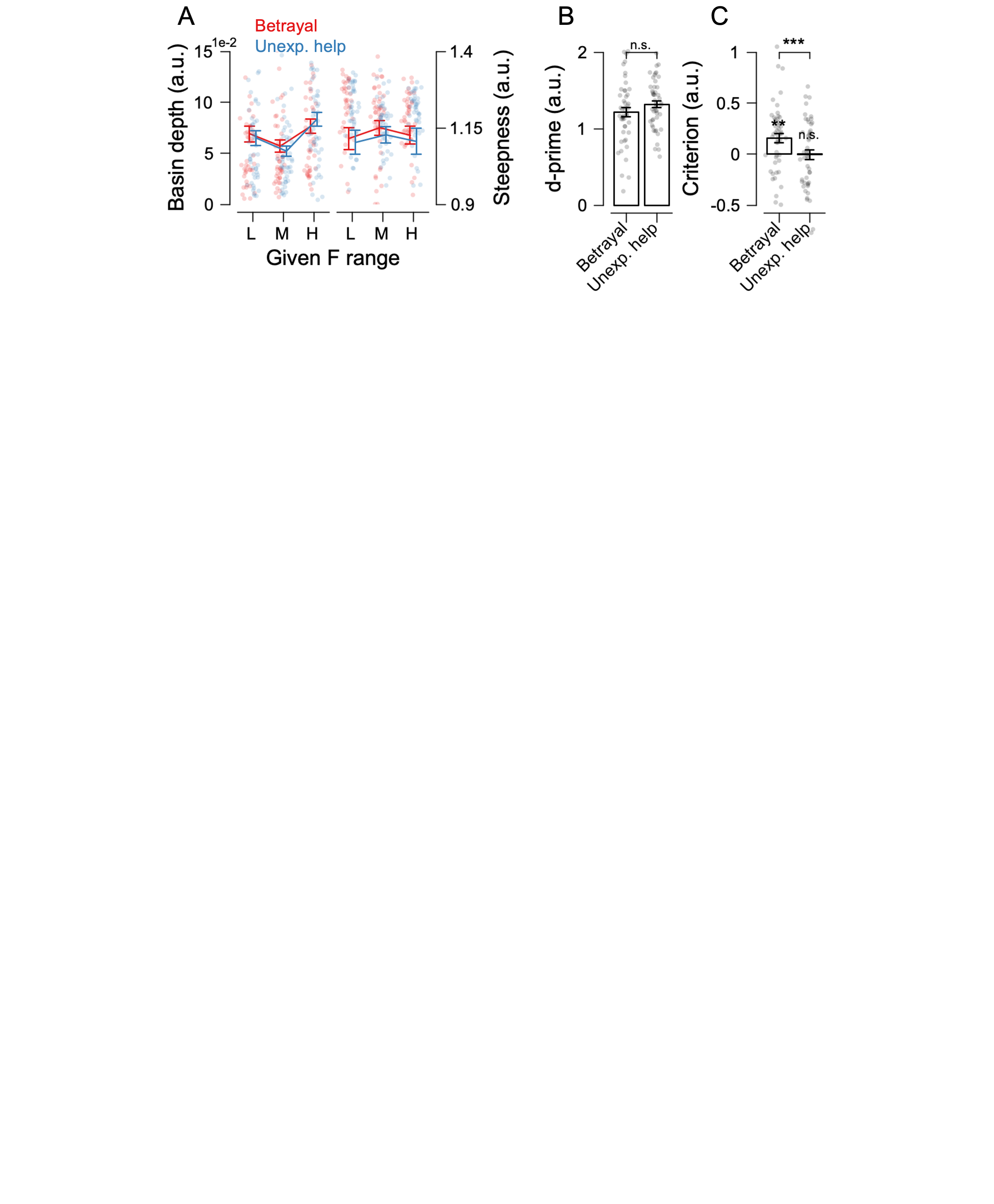

Supplement: S6 Fig — (A) The depth (difference between minimum and maximum values) and steepness (slope) of the inference energy landscape were similar across the basins of betrayal and unexpected help. (B) d′ values did not differ across conditions. (C) The decision criterion was significantly more conservative after betrayal. Error bars represent ±1 SEM, and dots represent individual participants. (TIFF) [file pcbi.1014200.s006.tiff]

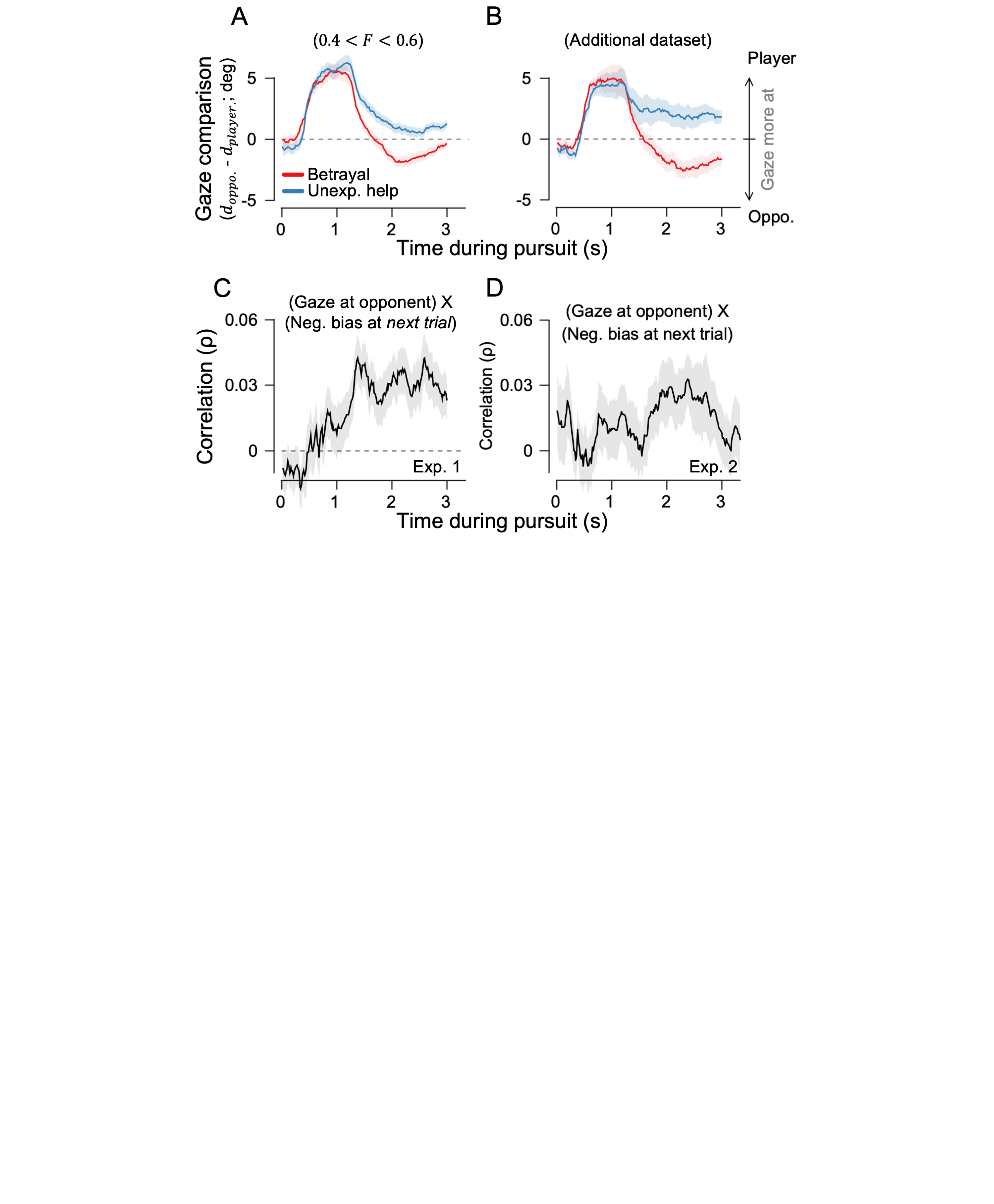

Supplement: S7 Fig — (A) Comparison of player and opponent gaze during the pursuit period, restricted to trials with matched F values (0.4 < F < 0.6). (B) Eight participants recruited in a separate session showed the same gaze-shift pattern. (C-D) Spearman’s correlation between gaze directed at the opponent and competitive bias in perceived F on the subsequent trial in Experiments 1 (B) and 2 (C). Error bars and shaded ribbons indicate ±1 SEM, and dots represent individual participants. (TIFF) [file pcbi.1014200.s007.tiff]

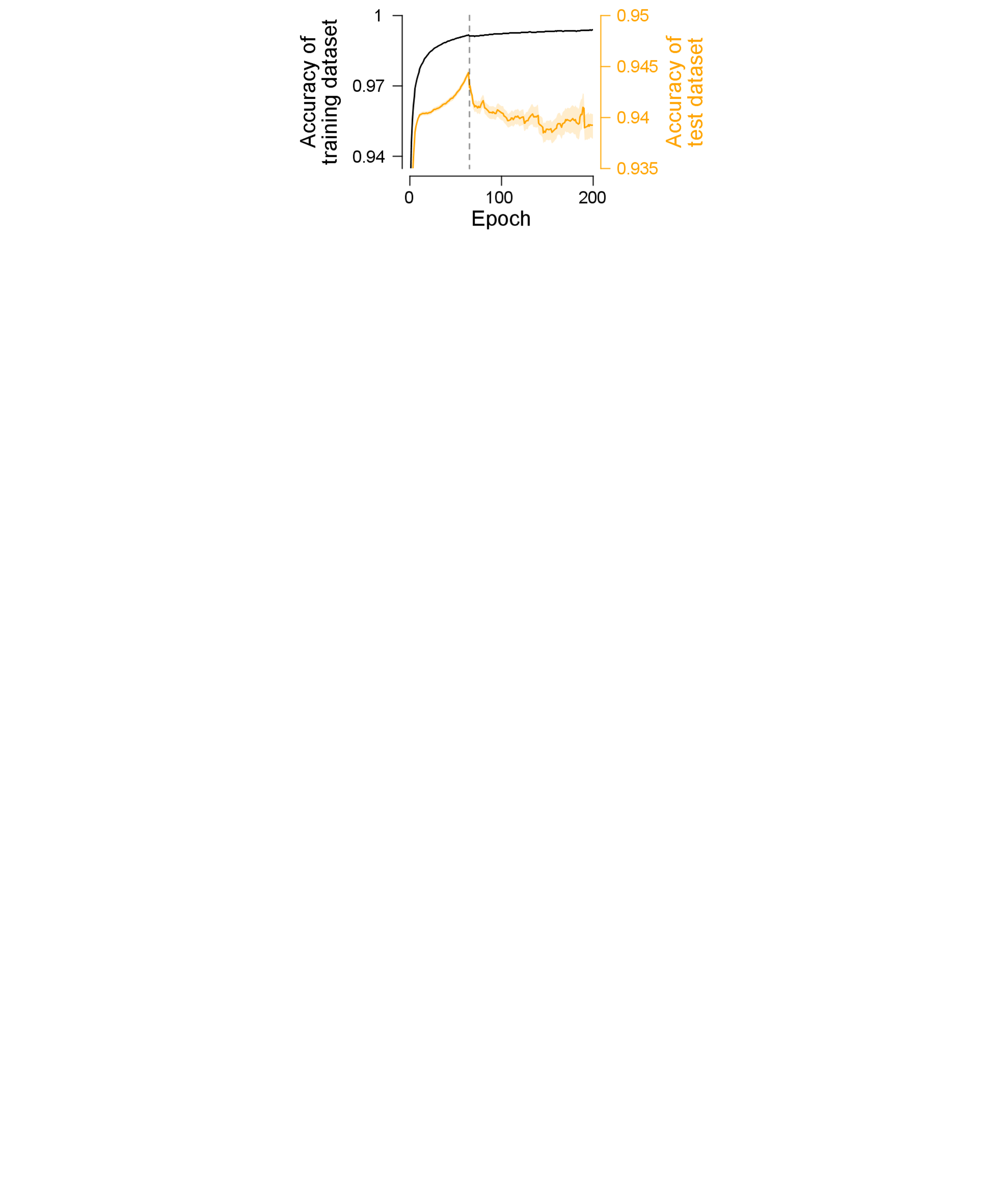

Supplement: S8 Fig — Black: training dataset (half of the dataset). Yellow: the test dataset (the other half). Shaded error bars represent ±1 SEM. (TIFF) [file pcbi.1014200.s008.tiff]

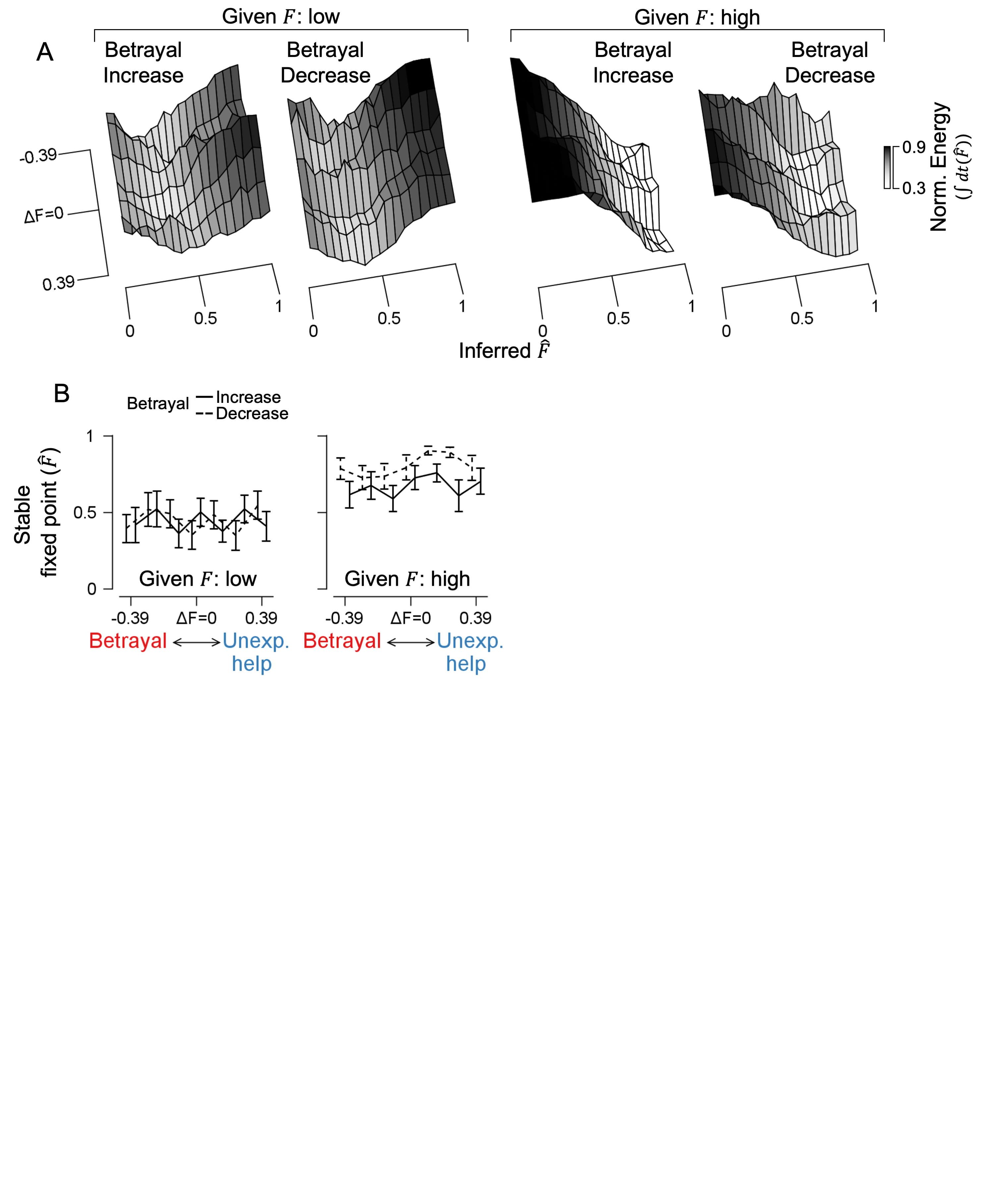

Supplement: S9 Fig — Separate energy landscapes are shown for trials with low (F < 0.33) and high F value (F > 0.67) (A), along with their corresponding stable fixed points (lowest points of the basins) in the phase diagram (B). Error bars represent ±1 SEM. Solid lines indicate the increasing betrayal phase, while dashed lines represent the decreasing phase. (TIFF) [file pcbi.1014200.s009.tiff]
