# Supplementary material for: Selective observation following betrayal shapes the social inference landscape
Source: PLoS Comput Biol. 2026 Apr 24;22(4):e1014200. doi: 10.1371/journal.pcbi.1014200 (PMC13193612; doi:10.1371/journal.pcbi.1014200)
Supplement: S1 Table — (DOCX) [file pcbi.1014200.s012.docx]

**S1 Table.**

| Regressor | | | Mean β | SEM | t-value (*t*_47_) | p-value |
| --- | --- | --- | --- | --- | --- | --- |
| Intercept | | | -0.742 | 0.147 | -5.006 | 0.000 |
| X_t_ | Player-Prey  distance | t1 | -0.006 | 0.002 | -2.739 | 0.009 |
|  |  | t2 | 0.002 | 0.004 | 0.465 | 0.644 |
|  |  | t3 | -0.002 | 0.005 | -0.324 | 0.747 |
|  |  | t4 | 0.013 | 0.005 | 2.886 | 0.006 |
|  |  | t5 | -0.022 | 0.002 | -9.689 | 0.000 |
|  | Opponent-Prey  distance | t1 | 0.020 | 0.002 | 7.991 | 0.000 |
|  |  | t2 | -0.025 | 0.005 | -5.032 | 0.000 |
|  |  | t3 | 0.020 | 0.005 | 4.272 | 0.000 |
|  |  | t4 | -0.027 | 0.003 | -8.387 | 0.000 |
|  |  | t5 | 0.021 | 0.002 | ​​10.539 | 0.000 |
|  | Player-Opponent  distance | t1 | -0.012 | 0.002 | -6.037 | 0.000 |
|  |  | t2 | 0.006 | 0.004 | 1.232 | 0.224 |
|  |  | t3 | 0.003 | 0.005 | 0.573 | 0.569 |
|  |  | t4 | 0.002 | 0.004 | 0.544 | 0.589 |
|  |  | t5 | 0.009 | 0.002 | 3.711 | 0.001 |
| ∆Fprev | | | 0.634 | 0.121 | 5.177 | 0.000 |

Logistic regression coefficients during Experiment 1. t1-t5 represent successive 200-ms time bins of the three-character trajectories.
