# Supplementary material for: Selective observation following betrayal shapes the social inference landscape
Source: PLoS Comput Biol. 2026 Apr 24;22(4):e1014200. doi: 10.1371/journal.pcbi.1014200 (PMC13193612; doi:10.1371/journal.pcbi.1014200)
Supplement: S2 Table — Inc and Dec refer to the betrayal-increasing and betrayal-decreasing phases, respectively. (DOCX) [file pcbi.1014200.s013.docx]

**S2 Table.**

| Regressor | | | Mean β | | SEM | | t-value (*t*_14_) | | p-value | |
| --- | --- | --- | --- | --- | --- | --- | --- | --- | --- | --- |
|  |  |  | Inc | Dec | Inc | Dec | Inc | Dec | Inc | Dec |
| Intercept | | | -0.616 | -0.430 | 0.232 | 0.185 | -2.561 | -2.236 | 0.024 | 0.044 |
| *X_t_* | Player-Prey  distance | t_1_ | -0.001 | -0.007 | 0.003 | 0.002 | -0.402 | -2.991 | 0.694 | 0.010 |
|  |  | *t_2_* | -0.012 | -0.006 | 0.006 | 0.004 | -1.970 | -1.430 | 0.071 | 0.176 |
|  |  | *t_3_* | 0.020 | 0.021 | 0.007 | 0.006 | 2.726 | 3.318 | 0.017 | 0.006 |
|  |  | *t_4_* | -0.008 | -0.002 | 0.006 | 0.005 | -1.170 | -0.403 | 0.263 | 0.693 |
|  |  | *t_5_* | -0.011 | -0.016 | 0.003 | 0.002 | -3.558 | -6.511 | 0.004 | 0.000 |
|  | Opponent-Prey  distance | t_1_ | 0.016 | 0.014 | 0.004 | 0.004 | 3.728 | 3.916 | 0.003 | 0.002 |
|  |  | *t_2_* | -0.016 | -0.019 | 0.010 | 0.005 | -1.568 | -3.528 | 0.141 | 0.004 |
|  |  | *t_3_* | 0.008 | 0.020 | 0.010 | 0.006 | 0.816 | 3.119 | 0.429 | 0.008 |
|  |  | *t_4_* | -0.015 | -0.026 | 0.006 | 0.005 | -2.604 | -4.579 | 0.022 | 0.001 |
|  |  | *t_5_* | 0.013 | 0.018 | 0.003 | 0.002 | 4.612 | 7.522 | 0.001 | 0.000 |
|  | Player-Opponent  distance | t_1_ | -0.012 | -0.010 | 0.004 | 0.004 | -3.205 | -2.583 | 0.007 | 0.023 |
|  |  | *t_2_* | 0.007 | 0.004 | 0.008 | 0.007 | 0.795 | 0.530 | 0.441 | 0.605 |
|  |  | *t_3_* | 0.001 | -0.001 | 0.008 | 0.007 | 0.086 | -0.137 | 0.933 | 0.893 |
|  |  | *t_4_* | 0.007 | 0.006 | 0.008 | 0.005 | 0.832 | 1.172 | 0.420 | 0.262 |
|  |  | *t_5_* | 0.004 | 0.007 | 0.004 | 0.003 | 0.931 | 2.524 | 0.369 | 0.025 |
| *∆F_prev_* | | | 1.160 | 1.422 | 0.324 | 0.219 | 3.453 | 6.245 | 0.004 | 0.000 |

Logistic regression coefficients during Experiment 2. t_1_-t_5_ represent successive 200-ms time bins of the three-character trajectories. Inc and Dec refer to the betrayal-increasing and betrayal-decreasing phases, respectively.
