# Supplementary material for: Selective observation following betrayal shapes the social inference landscape
Source: PLoS Comput Biol. 2026 Apr 24;22(4):e1014200. doi: 10.1371/journal.pcbi.1014200 (PMC13193612; doi:10.1371/journal.pcbi.1014200)
Supplement: Attachment — Submitted filename: response_to_reviewer.docx [file pcbi.1014200.s015.docx]

Reviewer #1: This study investigated how the choice of observational target biases inference about others' intentions. The authors showed that participants were able to modulate their behavior depending on their inference about the opponent's strategy (cooperative or competitive). Furthermore, this inference was biased when the opponent had acted more competitively than expected in the previous interaction. Using eye-tracking, the authors also found that the bias became stronger when participants chose to observe the opponent instead of their own avatar and this choice came at the cost of reduced control over their own avatar. Finally, they reproduced the behavioral patterns using a recurrent neural network (RNN) model.

We sincerely appreciate the thoughtful and constructive comments from all reviewers, which have greatly improved our manuscript. For clarity, text highlighted in blue in both this response letter and the revised manuscript indicates analyses and interpretations added in response to the reviewers’ suggestions.

I am wondering whether the study adequately addresses the main research question: How does choosing what to observe bias inference about others' intentions? (e.g., lines 17–18). The authors clearly demonstrated that participants' decision-making about whether to boost or hinder the opponent's speed (P(boost)) was modulated by the opponent's intention (F) to cooperate or compete. However, this promising finding is not directly related to the allocation of limited observational resources (i.e., selective observation).

The eye-tracking data may be somewhat relevant to the main research question. However, the corresponding description (lines 240–252) is overly speculative. That is, the conclusions drawn are not directly supported by the data and rely heavily on speculation. I do not question the quality of the data, but it remains unclear how the observed gaze patterns are related to the biased inference about others' intentions and social decision-making.

Thank you for raising this important question. We view this concern as consisting of two parts. The first is whether observation is indeed limited, and the second is whether factors other than selective observation could account for the observed changes in inference.

First, our results show that observation is costly. Our data demonstrate that shifting gaze away from the player’s avatar led to a lack of motor control (Fig 4E; Körding & Wolpert, 2004, PNAS). Since a similar concern was also raised by Reviewer 4, we conducted additional analyses showing that such gaze shifts led to lower rewards (Fig 4F). Together, these findings indicate that observation incurs a cost and is therefore selective, rather than being freely available.

Second, because Reviewer 3 also raised the possibility of additional psychological factors, we addressed these concerns in the revised manuscript by ruling out alternative explanations that could bias inference. We found no effect of monetary reward gain or loss (Fig 2G), psychological factors (e.g., loss aversion; S2A Fig and S2B Fig), response biases (S2C Fig), basic kinematic features (S3 Fig), or observation during the inference phase (Fig 4B). None of these factors accounted for the observed inferential bias. Instead, the only factor that differed between conditions and correlated with the magnitude of inferential bias was selective observation during the pursuit phase. Together, these results suggest that inference changed as a consequence of paying an observational cost, specifically the loss of motor control associated with shifting observational focus.

This aspect is now detailed further in the Results section as follows:

**[Line 305-309]** During the pursuit phase, participants needed to precisely control their own avatar to catch the prey, not monitor the opponent's movements. In fact, the more they looked away from their own avatar, the less stable their movement became (Fig 4E; *t*_47_ = 27.772, *p* = 6.200e^-26^), and rewards decreased accordingly (Fig 4F; *t*_47_ = -14.783, *p* = 2.701e^-19^).

**[Line 173-194]** One possibility is that betrayal caused greater monetary loss than the gain from unexpected help. However, when losses and gains were computed as the difference in reward between boost and hinder decisions within each condition, we observed the opposite pattern: the gain was larger in the unexpected help condition (Fig 2G; betrayal VS. unexpected help, *t*_47_ = 6.276, *p* = 1.028e^-7^). This indicates that reward value differences do not drive the asymmetry. Other factors, such as loss aversion [[22]](https://paperpile.com/c/sgWNBC/Ic4vZ), could also contribute to asymmetries in intention inference. However, perceived F values did not vary with reward magnitude (S2A Fig; slope, *t*_47_ = 1.237, *p* = 0.222), and the same result held even when we compared the trials with smaller and bigger rewards (S2B Fig; lower- vs higher-reward, *t*_47_ = -0.802, *p* = 0.427). We also considered whether participants strategically manipulated their reports. If so, such manipulation should be least feasible when perceptual evidence is near threshold. Yet even when the change in *F* was within half of the just noticeable difference (JND), responses still differed reliably between betrayal and unexpected-help conditions (S2C Fig; betrayal VS unexpected-help, *t*_47_ = 4.208, *p* = 1.150e^-4^ ), arguing against a conscious-response strategy as the primary driver of the asymmetry. We next asked whether the opponent’s observable behavior differed across conditions on the subsequent trial, which could provide an external cue for differential updating. Basic kinematic and geometric features, including agents’ positions, speed, acceleration, and angle relationships, were indistinguishable following betrayal versus unexpected help (S3 Fig). Thus, participants arrived at different decisions despite closely matched sensory evidence, suggesting that history primarily altered internal computations rather than the stimulus statistics.

**[Line 288-296]** Because the inference phase provides the most direct informational basis for the upcoming decision and imposes minimal motor demands, we first examined gaze allocation during this phase. And we found that gaze patterns were highly similar across conditions (Fig 4A). A ternary plot of gaze allocation during the inference phase showed that the mean gaze location was centered among the three agents (Fig 4B, top). When the analysis was restricted to a direct comparison between the player’s avatar and the opponent, the relative distance between gaze location and each agent likewise did not differ across conditions (Fig 4B, bottom; cluster-based permutation test, betrayal vs. unexpected help, all time points p > 0.05).

The authors used P(boost) as a proxy for the perceived intention of the opponent, inferred from observing their actions. Based on the result that P(boost) was modulated by F, they conjecture that participants inferred the opponent's intention. However, I wonder whether and how they can rule out alternative explanations. For example, participants may have inferred the opponent's ability to catch the prey, specific actions, or other attributes rather than intention per se.

Participants might infer the opponent's F value from specific behaviors in a given situation—for example, when the opponent runs straight toward the prey, a lower F value is likely. However, this alone cannot explain why perceived F values diverge after betrayal versus unexpected help. The basic physical properties of the characters' movements did not differ meaningfully between these two conditions (figure below, left), addressing the reviewer's suggestion that participants might instead be inferring the opponent's ability to catch the prey based on simple kinematic cues. If participants relied only on the opponent's immediate behavior to estimate F, the two conditions should not produce different inferences. Instead, as we argue, such differences are difficult to explain without considering that selective observation in the previous trial altered the structure of their inference process.

To make this point clearer for readers, we have added this explanation to the Results section as follows.

**[Line 188-194]** We next asked whether the opponent’s observable behavior differed across conditions on the subsequent trial, which could provide an external cue for differential updating. Basic kinematic and geometric features, including agents’ positions, speed, acceleration, and angle relationships, were indistinguishable following betrayal versus unexpected help (S3 Fig). Thus, participants arrived at different decisions despite closely matched sensory evidence, suggesting that history primarily altered internal computations rather than the stimulus statistics.

Likewise, although the authors claim that an opponent's behavior in the previous interaction biases social inference about their intention, an alternative interpretation is that participants' reaction to the inferred intention—rather than the inference itself—was biased. Is it possible to rule out this interpretation?

We also examined the possibility that participants might have deliberately chosen a specific response, rather than relying on genuine inference. To rule this out, we analyzed the Just Noticeable Difference (JND), the perceptual threshold for distinguishing the experimental conditions. If participants had been consciously aware of the F-value changes and deliberately biased their responses, they could not have produced such bias between the two conditions within the JND range. However, responses still systematically differed between the two conditions, even in the most ambiguous central region (where the stimulus difference was below half of the measured JND; figure below). This result supports the idea that participants' behavior was based on their inference, rather than intentional response distortion.

This analysis is now incorporated into the revised Results section as follows:

**[Line 182-188]** We also considered whether participants strategically manipulated their reports. If so, such manipulation should be least feasible when perceptual evidence is near threshold. Yet even when the change in *F* was within half of the just noticeable difference (JND), responses still differed reliably between betrayal and unexpected-help conditions (S2C Fig; betrayal VS unexpected-help, *t*_47_ = 4.208, *p* = 1.150e^-4^ ), arguing against a conscious-response strategy as the primary driver of the asymmetry.

Ideally, preregistration would have been conducted. This is not to suggest that exploratory analyses are inherently problematic. Moreover, it is unclear whether the authors are conducting exploratory or confirmatory analyses. If the analyses are confirmatory, preregistration would have been important; if exploratory, it would be valuable for the authors to clearly state this.

We appreciate the reviewer's concern regarding the potential ambiguity of our analysis. We confirm that this study was not pre-registered, and we explicitly state this fact in the Methods section of the revised manuscript as follows:

**[Line 513-514]** This study was not preregistered, and the analyses were exploratory and conducted after data collection.

To further address the reviewer's concerns regarding the robustness and replicability of our findings, we collected additional data from eight new participants and conducted the same Experiment 1. The key effects—(1) lower perceived F values following betrayal compared to unexpected help, (2) the shift in the energy landscape, and (3) the observational focus shift toward the opponent during betrayal—were all replicated in this additional dataset (Figure below). These new data have been incorporated into the revised manuscript. We hope that this supplementary evidence helps alleviate the reviewer's concerns.

It is also unclear how the RNN analysis contributes to understanding the underlying mechanism. While it is not surprising that a complex model like an RNN can reproduce behavioral data, it remains unclear what kind of understanding or interpretation this provides. Does it help constrain specific interpretations? For example, the statement "These RNN results demonstrate that reallocating observational resources to the opponent's movement mainly drives the inferential bias toward negative F values, revealing the source of the asymmetrical bias observed in human participants" is plausible. However, while this may be a relevant interpretation, can alternative interpretations be excluded? Given the complexity of RNN, there might be so many possible architectures that can reproduce the data. Why and how did the authors choose the specific one? Moreover, I do not agree with the authors' claim that RNN modeling can test causality (lines 100–102 and 292). The authors should carefully distinguish between causality in RNN simulations and causality in human decision-making.

Here, the goal of our RNN analysis was to strengthen the link between selective observation and inference. For this reason, we deliberately chose the simplest possible structure—a single-layer, fully connected neural network—to minimize unnecessary assumptions. Specifically, using the RNN, we sought to gain additional insight into two aspects. First, we examined how observation-related units—serving as a proxy for neural systems—interact with inference-related processes. Second, we tested whether changes in inference would emerge when the target of observation is experimentally controlled.

Addressing the first point, we explain why the inferential bias is asymmetrical: changes in observation-related units that selectively weight the opponent induce inferential bias, whereas corresponding changes in units weighting the player do not (Fig. 4F). Addressing the second point, altering the observational target—from the opponent to the player—was sufficient to neutralize the bias (Fig. 4H). Together, these results complement the human behavioral findings and provide additional insight into how inferential bias may arise—and how it may be overcome—through changes in observational allocation.

This aspect is now detailed further in the Results section as follows:

**[Line 349-368]** Why does bias emerge selectively when the network learns from betrayal? In our task, the latent variable F parametrizes the opponent’s intention, so the most diagnostic evidence for F should reside in the opponent’s movements, not in the player’s movement. This means that players can learn negative inferential bias from a betraying opponent’s movements and, in principle, positive bias from an unexpectedly helpful opponent. However, because selective observation of the opponent increases only in betrayal contexts, bias learning becomes asymmetric: observing betrayal strengthens negative bias, whereas overlooking unexpected help prevents positive bias from forming. Consistent with this prediction and with participants’ observation patterns, the input-weight updates from step 1 to step 2 showed that betrayal-sensitive networks selectively strengthened opponent-related weights, whereas unexpected-help-sensitive networks primarily strengthened player-related weights (Fig 5E). Crucially, across simulations, the magnitude of opponent-related weight changes (vector norms) tracked the degree of negative bias in inferred F, whereas comparable changes in player-related weights were largely unrelated to inferential bias (Fig 5F; ρ = 0.034 for player weights with p > 0.05 and ρ = −0.240 for opponent weights, *p* = 3.762×10⁻²¹). Together, these results suggest that betrayal-driven learning biases inference by concentrating learning on the opponent channel, which carries the most informative signals about latent intent in competitive contexts. As a result, competitive opponent kinematics exert a disproportionate influence on inferred F, whereas learning that prioritizes the player channel primarily improves control without systematically shifting the inferred intention.

In lines 157–161, the authors compared the magnitudes of monetary gain and loss, and concluded that betrayal did not cause greater monetary loss than the gain from unexpected help. However, psychological impacts of monetary loss can outweigh gains, as suggested by Prospect Theory in behavioral economics.

We thank the reviewer for the insightful comment. It is well-known that people experience a greater psychological effect from losses than from equivalent gains (loss aversion), and this phenomenon may have potentially influenced the results of the current experiment. Therefore, we plotted the perceived F value against the amount of money earned per trial (normalized from 0 to 1 for each participant). If a psychological effect, such as loss aversion, caused a greater sensitivity to losses, the perceived F value should decrease more steeply and asymmetrically when earnings approach 0 (loss situations) compared to when earnings approach the maximum (gain situations). However, the actual data analysis revealed that earned money did not affect the perceived F value (See left figure below; the slope of the curve was not significantly different from zero; t_39_=1.1586, p=0.254). Furthermore, when comparing the effect on F value changes on both sides of the median earned amount (loss/gain), no significant difference was observed (See right figure below; t_39_=1.152, p=0.256). This demonstrates that participants' inferential bias about the opponent's F value was not driven by psychological factors.

Furthermore, our RNN model reproduced human-like inference patterns solely from observational bias, without incorporating any psychological factors. This finding suggests that, in the context of our experiment, people's judgments about others' intentions were primarily driven by simple observational patterns rather than complex psychological effects.

We have now incorporated the relevant analysis into the Results sections as follows:

**[Line 178-182]** Other factors, such as loss aversion [[22]](https://paperpile.com/c/sgWNBC/Ic4vZ), could also contribute to asymmetries in intention inference. However, perceived F values did not vary with reward magnitude (S2A Fig; slope, *t*_47_ = 1.237, *p* = 0.222), and the same result held even when we compared the trials with smaller and bigger rewards (S2B Fig; lower- vs higher-reward, *t*_47_ = -0.802, *p* = 0.427).

How were the sample sizes in Experiments 1 and 2 determined?

Experiment 1 collected a sufficient number of participants to ensure statistical consistency (48 participants). Experiment 2 used a slightly modified design to replicate and extend the key findings of Experiment 1. The critical effects observed in Experiment 1—such as the asymmetrical inference bias following betrayal—were clearly and reliably reproduced in the new dataset. In addition, we recruited additional participants in Experiment 1. Notably, even with a smaller sample size in additional Experiment 1 (N = 8) than in Experiment 2 (N = 14), we were able to replicate the main results. This confirms that our findings are robust and not strongly dependent on sample size, but instead reflect a consistent effect.

Regarding reward payment, why was the original score multiplied by the opponent's F value?

Some participants exhibited poor motor control skills. During pilot testing, even when the opponent had relatively good intentions (e.g., F=0.7), participants sometimes lost the prey due to their own poor control. In these situations, participants tended to hinder the opponent and hunt the prey themselves, regardless of the opponent's good intentions. For this reason, we implemented an indirect reward for a 'good' opponent in the current experiment, as this approach allowed us to maintain consistent control over factors such as improvements in participants' avatar manipulation skills.

It would be helpful if the authors provided detailed information about the cluster-based permutation approach used to correct the p-value criterion.

We appreciate the reviewer's suggestion. We have now incorporated the relevant part into the Method sections as follows:

**[Line 880-884]** Specifically, this method grouped neighboring time points that showed similar effects into clusters, and a cluster-level t-value was calculated. We then shuffled the condition labels many times and recomputed these statistics to create a permutation distribution. By comparing the observed cluster to this distribution, we determined whether the effect was significant while properly controlling for multiple comparisons.

Is Fig. 1 directly relevant to the specific context of this study?

Fig 1. illustrates the central theme of this study. It emphasizes that information is recognized only through observation, forming a recurring cycle that links perception to subsequent action. Furthermore, as participants cannot see all information, the figure highlights that participants must selectively choose what to observe from various factors, including information about themselves, others' actions, and others' observations. Therefore, the figure provides an overview of the core concept of this study: how selective observations made in the past shape one's present inferences about others.

—————————————————————————————————————————

Reviewer #2: This manuscript developed an interactive prey-pursuit task where participants needed to infer an opponent's hidden intentions through observation to make decisions. They found that participants' behavior was history-dependent, where they overestimated the opponent's competitiveness after the opponent acted more competitively than expected, but no such bias when the opponent was more cooperative than expected. Further, the effect of betrayal history was greater when betrayal was an increasing phase than when it was a decreasing phase. To understand the bias, they used eye-tracking and found different observing patterns when participants experienced betrayal and unexpected help. Then they trained a task-optimized RNN and showed that shifting the focus of observation can reverse inference biases.

This paper is an insightful contribution to the field. The authors proposed a novel task which allowed inferring others' intentions through a dynamical, continuous process and real-time interaction, which has potential to explore a broad range of social problems such as coordination and game. And they introduced the energy landscape framework, which provided new insight for understanding decision-making process. Their results are comprehensive. However, we have identified several issues that need to be addressed to strengthen the results and clarify the conclusions. Further details on our concerns can be found below.

We appreciate the reviewer's insightful feedback, which significantly strengthened the manuscript. For clarity, we note that the text highlighted in blue in both this response letter and the revised manuscript corresponds to analyses and interpretations added in response to the reviewers’ suggestions.

1. The authors defined perceived/inferred friendliness as the estimated choice probability by autoregressive logistic regression, and most of the results were based on this index. But the regression details are missed, including the precise regression equation, detailed regression results (table with regressors and the corresponding statistics), and estimation/optimization details. They are important to support the model. Besides, whether the results remain unchanged when using real boost probability instead of the regression estimation? At least some model-free results should be shown to validate the conclusions and analyses.

The logistic model aims to capture time-series data reflecting how participants' inference evolves over multiple time points; however, direct comparison with the final behavioral choice at a single point is not directly comparable.

Nevertheless, similar conclusions can be drawn indirectly using the Signal Detection Theory. In SDT, the 'd-prime' value (sensitivity of choice) is analogous to the depth of the 'energy landscape' that leads to a conclusion. A deeper landscape leads to faster and more accurate convergence (higher d-prime), while a shallower one leads to slower convergence (lower d-prime). On the other hand, a change in the 'criterion' can be interpreted as a shift in the basin of the energy landscape. Consistent with our model analysis results, where the 'depth of the landscape' remained unchanged but the 'stable fixed point' shifted, the SDT analysis showed no difference in 'd-prime' values across conditions but did reveal a change in the 'criterion' (see figure below).

Additionally, to enhance the transparency of our model, we have incorporated a detailed explanation of the regression model, as suggested by the reviewer (see S1 Table).

The consistency between these two analyses provides a more comprehensive view for readers; therefore, we have included this finding in the revised manuscript as follows:

**[Line 273-283]** Because basin geometry and basin position have direct behavioral analogs, with basin depth and steepness relating to sensitivity (d′) and basin position relating to decision criterion, we next tested these landscape-derived predictions using Signal Detection Theory. There were no differences in the sensitivity across conditions (S6B Fig; betrayal VS. unexpected help, *t*_47_ = 1.827, with *p* = 0.074). Instead, the decision criterion differed markedly across conditions, remaining near zero after unexpected help but shifting to a significantly positive value after betrayal (S6C Fig; betrayal: *t*_47_ = 3.336, *p* = 0.002; unexpected-help: *t*_47_ = 0.083, *p* = 0.934; betrayal vs. unexpected-help: *t*_47_ = -4.070, *p* = 1.788e^-4^). This result corresponds to the energy landscape results, indicating that after betrayal, participants adopted a more conservative criterion and required clearer cooperative behavior before selecting an option to boost the opponent.

2. Did the patterns of response time during decision making have any differences after experiencing betrayal or unexpected help under different F levels? And the relationship between response time and perceived F is also needed to be checked.

Thank you for the suggestion. In addition to the relationship between decision time and the F value (S4B Fig), we also examined the relationship between decision time and the bias in F (figure below). We found no meaningful trend between the two variables.

This result has been included in the Results section as follows:

**[Line 207-209]** Additionally, decision times were similar across conditions (S4A Fig; betrayal vs. unexpected help, all p > 0.05), and they were not associated with the magnitude of the inferential biases (S4B Fig; *t*_47_ = 1.490, *p* = 0.143), [...]

3. Lack of formal specification (mathematics) of the energy calculation and RNN loss function.

Thank you for pointing this out. In the revised manuscript, we have added explicit equations and detailed descriptions for both the energy landscape calculation and the RNN loss function, so that readers can clearly follow the computational steps involved as follows:

**[Line 747-749]** Specifically, we reconstructed the energy landscape U(F̂) as

$U\left( \hat{F} \right) = \int g\left( \hat{F} \right)d\hat{F}'$ (where $g\left( \hat{F} \right) = \left| \frac{d\hat{F}}{dt} \right|_{\hat{F}}$)

**[Line 813-814]** The total MSE in the first step was computed as

*MSE = ∑_trial_ Loss^2^* where *Loss = F_predict_ - F_actual_*

**[Line 824-829]** As a result, the F̃ value inferred by the RNN deviated from the given *F* value in the corresponding inference phase. This deviation yielded MSE loss from a negative difference in the betrayal trials (Loss^−^ = *F_predict_ - F_actual_ < 0*) or a positive difference in the unexpected-help trials (Loss^+^= *F_predict_ - F_actual_ > 0*). The total loss from 200 trials was computed as follows;

$MSE = \beta_{bet}\cdot{Loss}^{-}+(1-\beta_{bet})\cdot{Loss}^{+}$

—————————————————————————————————————————

Reviewer #3: Summary

In this study, the authors introduce a novel two-agent hunting paradigm to investigate how inferences about others' motives are shaped by experience and selective observation. They found that interacting with opponents that behaved more competitively than expected selectively and negatively biased subsequent inferences regarding their cooperativeness. Eye gaze analyses suggest that subjects allocated more attention to the other's actions during such betrayals, which in turn was linked to increased bias in subsequent trials. They further characterized this bias with an energy landscape approach, and used an RNN to claim a causal effect of attention on the bias. In a second experiment, they provide evidence that this effect propagates across trials in a hysteresis-like fashion.

The manuscript addresses a relevant question using an innovative task and an impressive array of analyses. The text is well-written, logically structured, and the large number of high-quality figures transparently portray the data and nicely illustrate all main analyses, which is really helpful. The findings are interesting and the conclusions are largely well supported by the data. However, three major points (and a few minor ones) should be addressed before publication: The results raise one main question that currently remains unaddressed; the RNN procedures seem to (at least in parts) predispose their outcomes; and some of the findings in Exp. 1 seem to be at odds with the conclusions from Exp. 2.

We appreciate the reviewer’s constructive comments. They have greatly helped us improve the manuscript. To aid readability, we indicate in blue the analyses and interpretations that were added in response to the reviewers’ suggestions in both this response letter and the revised manuscript.

Major comments

#1) Given the center-stage and novelty of the asymmetric inference bias, a central question that surprisingly remains unaddressed in results and discussion is where this bias originates. While the authors rule out larger monetary consequences as a driver (via the analysis in Fig 2G), could loss aversion change this conclusion? Relatedly, could betrayal aversion or similar psychological motives be at play? On the other hand, the fact that the RNNs seem to recreate the asymmetry speaks to reasons other than psychological ones. Can the authors provide any such reasons, or at least speculate about them?

We thank the reviewer for the insightful suggestion. As pointed out, we investigated whether changes in participants' psychological states in response to reward loss and gain might have influenced their responses. However, given two key findings—first, that the probability of choosing the 'boost' item did not change significantly with reward level, and second, that our RNN model (which lacks psychological factors) displayed the same inference pattern—we concluded that the influence of the psychological effect on the response did not confound our results.

We have incorporated the corresponding analysis into the Results as follows:

**[Line 173-182]** One possibility is that betrayal caused greater monetary loss than the gain from unexpected help. However, when losses and gains were computed as the difference in reward between boost and hinder decisions within each condition, we observed the opposite pattern: the gain was larger in the unexpected help condition (Fig 2G; betrayal VS. unexpected help, *t*_47_ = 6.276, *p* = 1.028e^-7^). This indicates that reward value differences do not drive the asymmetry. Other factors, such as loss aversion [[22]](https://paperpile.com/c/sgWNBC/Ic4vZ), could also contribute to asymmetries in intention inference. However, perceived F values did not vary with reward magnitude (S2A Fig; slope, *t*_47_ = 1.237, *p* = 0.222), and the same result held even when we compared the trials with smaller and bigger rewards (S2B Fig; lower- vs higher-reward, *t*_47_ = -0.802, *p* = 0.427).

#2) If I understand the RNN analyses correctly, the way the training is set up in step 2 explicitly trains the models to make asymmetrically biased judgments. Specifically, if the loss used for training is the difference between the F value as predicted by the RNN (for the input sequence), and the value from the corresponding inference phase (which does not seem to enter the model again and hence is not available to the network), the *only* way to improve the prediction is by introducing a bias. As this can't work for an equal number of positive and negative prediction error trials, the network is forced during training to bifurcate to only focus on one type of errors (via adjusting the beta parameter). Is this understanding correct? If so, the authors should revise the corresponding sections to better reflect this. However, even if it is the case (or possibly even more so), it's still interesting and unclear why the bias only seems to emerge in the betrayal nets (which relates to #1 above).

The goal of the second step of RNN training was to observe how the system, learned in the first phase, resolves the discrepancy in F values that arises during betrayal and unexpected help situations. During this process, as the reviewer pointed out, once a bias begins to form, it is likely to drift toward a particular direction.

We also revised the manuscript to explain why changes in the opponent-related input weights have a disproportionately larger impact on the inferred F value (Fig 4F). Because F reflects the opponent's latent intention, its estimation relies primarily on the opponent's movement information rather than the player's or the prey's. As a result, modifying the input weights associated with the opponent alters the inferred F far more strongly than modifying the weights related to other inputs.

To quantify this effect, we computed the vector norms of the input weight changes for the player- and opponent-related channels as the network adjusted from step 1 to step 2. We examined how these changes influenced the inferred F value (Figure below). The analysis showed that changes in the opponent-related input weights induced a clear negative bias in F. In contrast, changes in the player-related input weights had little to no impact. This demonstrates that the network's inference is structurally anchored to opponent information and supports our central claim that selective observation of the opponent has a stronger influence on subsequent inference.

We have incorporated the corresponding analysis into the Results as follows:

[Line 349-368] Why does bias emerge selectively when the network learns from betrayal? In our task, the latent variable F parametrizes the opponent’s intention, so the most diagnostic evidence for F should reside in the opponent’s movements, not in the player’s movement. This means that players can learn negative inferential bias from a betraying opponent’s movements and, in principle, positive bias from an unexpectedly helpful opponent. However, because selective observation of the opponent increases only in betrayal contexts, bias learning becomes asymmetric: observing betrayal strengthens negative bias, whereas overlooking unexpected help prevents positive bias from forming. Consistent with this prediction and with participants’ observation patterns, the input-weight updates from step 1 to step 2 showed that betrayal-sensitive networks selectively strengthened opponent-related weights, whereas unexpected-help-sensitive networks primarily strengthened player-related weights (Fig 5E). Crucially, across simulations, the magnitude of opponent-related weight changes (vector norms) tracked the degree of negative bias in inferred F, whereas comparable changes in player-related weights were largely unrelated to inferential bias (Fig 5F; ρ = 0.034 for player weights with p > 0.05 and ρ = −0.240 for opponent weights, *p* = 3.762×10⁻²¹). Together, these results suggest that betrayal-driven learning biases inference by concentrating learning on the opponent channel, which carries the most informative signals about latent intent in competitive contexts. As a result, competitive opponent kinematics exert a disproportionate influence on inferred F, whereas learning that prioritizes the player channel primarily improves control without systematically shifting the inferred intention.

#3) The findings from Exp. 2 are summarized to suggest that "stopping the betrayal will not immediately restore neutral inference due to its momentum [when the degree of betrayal continues to increase]." However, in Exp. 1, the authors report: "[...] repeated betrayal progressively reinforced the bias toward more competitive inferences (S1D Fig; linear trend analysis [...]). However, the biases after the betrayal experience immediately returned to a neutral state when the betrayal ceased (i.e., at block change) (S1E Fig)." How can these two findings be reconciled - shouldn't momentum in Exp. 1 prevent an immediate return to a neutral state (even if it's not increasing as in Exp. 2)?

Thank you for raising this important question. The key to reconciling the findings from Experiments 1 and 2 lies in how gradual betrayal changes.

In Experiment 2, the amount of betrayal changed gradually across trials, and we found that inference did not immediately return to a neutral state, even when betrayal began to subside, reflecting momentum in the inference dynamics. In contrast, Experiment 1 involved a discontinuous block transition in which unexpected help replaced betrayal, and the inference bias disappeared rapidly (S1E Fig). This indicates that recovery from inferential bias depends on such discontinuous shifts.

Thus, the immediate recovery observed in Experiment 1 does not contradict the momentum effect identified in Experiment 2. Rather, the two experiments together show that inferential momentum persists under gradual changes in betrayal, but can be overcome by abrupt transitions—either in the opponent’s behavior or, as demonstrated in our RNN simulations, by a forced reallocation of observational focus (Fig. 5H).

We have now incorporated the corresponding logics into the Results as follows:

**[Line 421-426]** This suggests that when the degree of betrayal continues to increase, inference does not immediately return to a neutral state due to its momentum, even if betrayal gradually subsides. Taken with Experiment 1, the present findings provide a more comprehensive account of the hysteresis-like behavior: its expression depends on the sharpness of the transition from betrayal to unexpected help, which in turn redistributes observational resources.

Minor comments

Choice of title/abstract: The authors report two main findings: Exp. 1, and the majority of analyses and figures, are concerned with characterizing the asymmetry of the history-dependent inference bias which results from the very last trial, while Exp. 2 is concerned with effects that propagate across trials in a hysteresis-like fashion. This makes one wonder if the title should really focus on the latter, as it currently does, rather than the former. Relatedly, the authors might consider mentioning the energy landscape and/or RNN analyses in the abstract, in order to attract the right target audience.

We have revised the title as “Selective Observation following Betrayal shapes the Social Inference Landscape” to reflect the reviewer's suggestion. Thank you for the helpful feedback.

Figure 2E: Is it possible that the top two panels are flipped? The top-left one looks as if the opponent is going straight for the prey (i.e., being competitive), whereas the top-right one looks more like herding.

We apologize for the confusion. We have changed the example in the revised manuscript to one that is clearer.

Figure 2F: "hindering a low-F opponent was more beneficial" - but hindering seems to generally just render the opponent's F inconsequential, as the reward does not seem to be affected by F (whereas the effect of boosting changes as a function of F); is this because the other's behavior simply becomes irrelevant when hindered?

Selecting “hinder” renders the computer opponent functionally incompetent, reducing its ability to influence the outcome of the game; however, the opponents still aim to intercept the prey competitively. Thus, when facing a low-F opponent, choosing to hinder becomes the relatively more advantageous option, even though the opponent continues to attempt to intercept the prey.

Figure 3A: Please state that the figure is across participants, and refer to the corresponding Supplementary Figure for individual subjects.

We revised the manuscript to clarify that Fig 3A displays the across-participant average, whereas Fig. S1A presents individual data.

Line 232: Please also report effect sizes in the main text (which are depicted in Figure 4C/D); especially given that they are rather small.

We also added the effect size as:

**[Line 311-313]** Longer viewing durations produced the comparable effect (S7B Fig; ρ = 0.032 ± 0.008, mean ± SEM; p = 0.010 from 1.25–1.80 s and p = 0.001 from 1.83–3 s after pursuit onset).

Since the F values were fully randomized across trials, the effect size is expected to be relatively small.

Line 307: "As in Experiment 1, stronger betrayal experiences in previous trials led to a greater bias toward" - is this dose-response relationship really shown? I thought Exp. 1 always compared betrayal vs. unexpected help at t-1, whereas Exp. 2 compared increasing vs. decreasing betrayal (which, if anything, rather suggests that stronger betrayal in previous trials, as experienced in the decreasing phase, lead to *smaller* biases).

We can reproduce a figure showing that gaze toward the opponent is directly correlated with the magnitude of negative bias in the next trial on a trial-by-trial basis (as in S7B Fig for Experiment 1, now applied to Experiment 2; see figure below). We previously omitted this result because it replicates a similar finding from Experiment 1. The figure is now included as S7C Fig in the revised manuscript.

The corresponding results are incorporated into the Results as follows:

**[Line 403-404]** The relationship between observation and inferential bias followed a similar trend as in the experiment 1 (S7C Fig).

Line 281: "These RNN results demonstrate that reallocating observational resources to the opponent's movement mainly drives the inferential bias", but before that it is stated that "a decrease of the input weight led to a corresponding inference bias". How can a *de*crease in weights be interpreted as allocating *more* resources?

Thank you for pointing out that our wording could be misleading. The "decrease in input weight" was intended to describe a directional change that enhances negative bias, rather than an actual reduction in input strength. Because we agree that this wording could also confuse readers, we have revised the figure (Fig 5E) to show the absolute change instead.

Relatedly, can the authors speculate on why more/less focus on the other (by humans and RNNs) only affects inferences regarding competitiveness? This relates to the major comment #1 above, but with emphasis on the causal effect on attention.

Since 'where to observe' often reflects 'where to attend', attention could also be a potential underlying mechanism. As in comment #1, we have expanded the discussion to help readers better understand this possibility as follows:

**[Line 495-497]** Moreover, since observational focus shifts are often closely coupled with changes in attentional allocation, future work should also incorporate the role of attention.

Typos/wording:

281: "F value" seems to be accidentally added.

626: "by following the steps [?] (Fig 5A)" end of the sentence seems to be missing.

652: "unexpected [help?] trials"

718: "During the inference phase [...]" -> "Across trials, the F value during the inference phase [...]" would be correct, as there is no change *during* the inference phase, right?

731: "resembles" -> "can be represented as" would be more appropriate.

733: "moving-windowed trajectories [during the inference phase]" would be more explicit.

Thank you for identifying our mistakes. The relevant parts have been corrected.

—————————————————————————————————————————

Reviewer #4: Son and Yoo have developed an interesting new behavioural task, in which hidden intentions of a computer opponent can be deduced (i) by past interactions and (ii) by selectively allocating one's eye gaze to the spatial trajectory of this opponent. Both participants' choices and their eye gaze were related to the fluctuating "friendliness" (i.e., cooperativeness) of the agent. The authors used an autoregressive logistic regression model and developed a RNNs to describe and mimic participants' behavior.

Specifically, participants (depicted as a green dot on a screen) could chase prey (depicted as a blue square). Crucially, an opponent (depicted as red dot) could help or hinder. The intention of the opponent varied from cooperative to competitive (manipulated via the parameter F for friendliness). Depending on the opponent's intention, participants should "boost" or "hinder" the opponent's speed in order to maximize their own probability to catch the prey. Each trial had two phases: Inference and pursuit. Unbeknownst to the participants, the intention of the opponent changed between these two phases resulting in two conditions: betrayal (i.e., cooperative to competitive) and unexpected help (i.e., competitive to cooperative). These two conditions changed probabilistically in a block-wise fashion in Experiment 1 (n=54) and in a "decreasing/increasing fashion" in Experiment 2 (n=14). Results showed overall lower boost decision after betrayal trials than after unexpected help trials. The inferred F (in regression models) differed from the true F in the betrayal trials but not the unexpected help conditions. Gaze on the opponent also differed between these conditions.

In my view, the paper is highly innovative and overall well-written. Please see below for my comments and questions.

We are grateful for the reviewer's insightful comments. They helped us to improve our manuscript significantly. For clarity, blue-highlighted text in both this response letter and the revised manuscript denotes analyses and interpretations added in response to the reviewers’ comments.

1. Trial & block structure & changes in F: This was not entirely clear to me and is also one of the reasons why I have tried to summarize the experimental setup in such detail above. A few related questions:

a. What was the motivation for changing F between inference and pursuit phases? Why was there an inference phase at all? That is, why exactly did the authors need a phase in which participants could not act? Could the change in F not be implemented ONLY between consecutive trials? [Asking the change was not across trial]

b. Crucially, why was there a block-wise change in the sign of the change of F? For many of the analyses it is unclear whether the authors looked at trial-by-trial differences or block-by-block differences. I think they mostly looked at the former but then why is it necessary to have block-wise changes?

We apologize for the confusion. The primary goal of this study was to investigate how participants respond when their expectations—regardless of the role—are violated. The inference phase was designed to allow participants to form a baseline expectation about the opponent’s role before making a decision. To prevent this baseline from being influenced by external factors, we separated the inference process from motor execution. If participants had been required to infer the opponent’s intention while controlling their avatar in the pursuit phase, differences in motor ability among individuals, for example, could have caused them to overestimate or underestimate the opponent’s F value. We thus manipulated the opponent’s behavior in the pursuit phase so that it violated this expectation in a specific direction—either as betrayal or unexpected help.

Since our main goal was to investigate the history-dependent effects of social role violations, a block structure was necessary to accumulate a minimal amount of history. If betrayal and unexpected help had alternated on a trial-by-trial basis, for instance, the effect of the current trial could have been confounded with the influence of the immediately preceding trial or even the trials before that. For these reasons, Experiment 1 used a block design, and Experiment 2 introduced gradual changes.

c. F changes quite quickly (Fig 2D upper part). Is this truly necessary? This makes learning very hard. What would the authors and what would their models predict for slower changes? It could be that the bias would disappear.

The goal of our study was to examine how participants respond when their expectations—regardless of the specific role—are violated. Accordingly, we randomly assigned F values on each trial, while manipulating ΔF either in a block-wise manner (Experiment 1) or gradually across trials (Experiment 2).

If we had instead designed the task such that the F value itself changed slowly over time, this would have allowed us to examine the interaction between the history of a particular role and violations of expectations about that role. While this is an interesting direction for future work, it falls outside the scope of the present study.

d. Crucially, the definition of betrayal vs. unexpected help conditions seems to only depend on the sign of the change in F between inference and pursuit phases but not the "ground level" of F in the inference phase. I would like to see some of the analyses that the authors did with deltaF with F_inference and F_pursuit as separate predictors.

Thank you for the suggestion. We separated ΔF into F_inference and F_pursuit in the logistic regression model and repeated the inference-landscape analysis. The results were broadly consistent with our original findings (figure below).

e. The authors should show the distribution of these F values. For example, Fig 3A shows binned data but it is unclear how many data points are within each bin. I guess the middle ranges of F contain more data points.

Since the F values were randomly assigned during the inference period, the number of data points within each bin is nearly identical. Please refer to the distribution of the given F values below.

2. The "energy landscape analyses" were also not quite clear to me:

a. The methods section has more details but the following description in the results is not quite enough for the results section. "Thus, the model incorporated two key parameters: βt, which captures how well participants infer the opponent's intentions from the trajectories of the three characters during the inference phase, and βhys, which regulates how past experiences of betrayal or unexpected help influence inference bias. In sum, βt primarily alters the steepness of the psychometric curve, while βhys primarily modulates the magnitude of inference bias (S2 Fig)." I found the following part in the methods helpful "To construct this, we computed the pairwise Euclidean distances among the three characters at each time point over a 1s period, then averaged them within 200-ms bins, resulting in 15 representative feature values (3 characters × 5 time points).

We revised the manuscript so that the Results section now contains a more detailed explanation of the model's architecture as follows:

**[Line 225-246]** We reconstructed the energy landscape by tracking how inference stabilized over time using an autoregressive logistic regression model (Fig 3D). This model captured how participants inferred the opponent’s intention moment by moment from the temporal patterns observed in the movements of the three characters. Specifically, during the final 1-second inference window, we computed the pairwise Euclidean distances among the three characters and averaged them in 200-ms bins, yielding 15 representative features that served as regressors. Thus, the 15 coefficients for this regressor, β_t_, reflect how accurately participants inferred the opponent’s intention from the trajectories of the three agents during the inference phase, and weakening β_t_ correspondingly attenuates this inference sensitivity (S5 Fig, compare the differences across rows). We also included the strength of betrayal or unexpected help experienced in the previous trial (∆F_previous_) as an additional regressor. The coefficient for this regressor, β_hys_, indicates how strongly past experiences shaped the magnitude of the resulting inference bias (S5 Fig, compare the differences across columns). S1 Table shows the fitted results of the regression model. By applying this procedure frame by frame for each trial, the model produced an inferred time series showing how participants’ estimates evolved toward their final decision for a given inference-phase movie clip. When the inferred time series converges toward the given F value over time, little change is observed near the center of the F range, whereas large fluctuations appear toward the extremes. To quantify this pattern, we computed the time derivative of the inference time series within each F-value bin and integrated it to estimate the degree of instability [[25]](https://paperpile.com/c/sgWNBC/xogBP). Based on this instability measure, we then reconstructed the inference landscape (see Materials and methods for details).

b. Also, Fig 3D does not quite make it clear. This is easy to improve.

We have revised Fig. 3D to provide a more intuitive visualization of the model's architecture, and we hope this makes the framework easier for the reviewer to understand.

c. Why did the authors not look into the three different trajectories for these analyses? Relatedly, we did they not look at the trajectory of the prey for the gaze data (see comment below)?

Thank you for this question. Since the task was to hunt the prey, it is natural that the participant observed the prey closely. Indeed, when we visualized gaze distance using a ternary scatter plot—constructed from the inverse distances between the gaze point and the three characters (averaged from 1.5 to 3 seconds after pursuit onset)—participants consistently fixated close to the prey (Figure below; each dot is an individual participant). However, the difference between the two conditions (Betrayal vs. Unexpected Help) did not depend on how closely they examined the prey. Instead, the key distinction lay in how gaze was distributed between the opponent and the player. For this reason, the original manuscript explained the effect using a simpler opponent-versus-player comparison. For an intuitive explanation, we added the ternary scatter plot and corresponding explanations into the manuscript as follows:

**[Line 291-293]** A ternary plot of gaze allocation during the inference phase showed that the mean gaze location was centered among the three agents (Fig 4B, top).

**[Line 298-300]** Gaze differed across conditions in whether it was biased toward the player or the opponent, whereas the extent of prey-directed gaze did not differ (Fig 4D, top).

d. Why are only three time points plotted in Fig 3E and not the 5 time points analysed?

We could certainly present the data at a finer temporal resolution (e.g., 0.5s intervals) or even as a continuous time series. We adopted the three-stage partitioning primarily to allow readers to visually gauge the temporal convergence of the psychometric curves relative to the raw data (Fig. 3A). Splitting the time points more or less did not change the overall meaning.

e. I did not understand the following: "To analyze the meaning of each regressor on intention inference, we conducted simulations by multiplying 0, 1/3, 2/3, or 1 on βt, or multiplying 0, 1, 2, or 3 on βhys."

We intended Figure S2 to provide a visual representation of the functional meaning of each regressor within the logistic regression model. To achieve this, after the initial model fitting, we selectively attenuated or amplified the contribution of a specific term (by multiplying its corresponding coefficient, β_t_ or β_hys_, by a specific factor). We then plotted the predicted outcomes of this modified model as a psychometric curve to illustrate the resultant effect. Recognizing that this key methodological detail was insufficiently detailed in the initial submission, we have now revised and expanded this explanation as follows:

**[Line 231-237]** Thus, the 15 coefficients for this regressor, β_t_, reflect how accurately participants inferred the opponent’s intention from the trajectories of the three agents during the inference phase, and weakening β_t_ correspondingly attenuates this inference sensitivity (S5 Fig, compare the differences across rows). We also included the strength of betrayal or unexpected help experienced in the previous trial (∆F_previous_) as an additional regressor. The coefficient for this regressor, β_hys_, indicates how strongly past experiences shaped the magnitude of the resulting inference bias (S5 Fig, compare the differences across columns).

3. Eye-tracking:

a. My main point here is what are the distances to the prey? Are some of these results spurious in the sense that participants focus more on the prey which is influenced by the F value of the opponent?

As the ternary scatter plot comparing the distance between the gaze location and the three characters illustrates, participants observed the prey closely. Crucially, however, the distance to the prey did not differ significantly between the two experimental conditions.

b. The correlations very small. Could the authors comment on this and try to situate the explained variance?

We agree that the correlation coefficients are consistent but modest. This is largely because the two variables used in the correlation come from different trials. Since the opponent’s F value varies randomly from trial to trial, this fluctuation introduces substantial variability into the measured bias, inevitably reducing the overall correlation. We now report the effect size for this analysis and have added this explanation to the revised manuscript as:

**[Line 311-313]** Longer viewing durations produced the comparable effect (S7B Fig; ρ = 0.032 ± 0.008, mean ± SEM; *p* = 0.010 from 1.25–1.80 s and *p* = 0.001 from 1.83–3 s after pursuit onset).

c. I am not quite convinced by the costs in terms of "jerk strength." Did this result in fewer catches of the prey?

Following the reviewer's suggestion, we analyzed the correlation between gaze distance between the player (averaged from 1.5 to 3s after pursuit stage onset) and the amount of reward (money) obtained in the current trial. Consistent with the indirect evidence provided by the jerk analysis, the results showed that closer gaze fixation on the player was associated with faster prey capture and, consequently, higher reward earnings.

We have now incorporated the corresponding logics into the Results as follows:

**[Line 307-309]** In fact, the more they looked away from their own avatar, the less stable their movement became (Fig 4E; *t*_47_ = 27.772, *p* = 6.200e^-26^), and rewards decreased accordingly (Fig 4F; *t*_47_ = -14.783, *p* = 2.701e^-19^).

d. The following section in the results seems to me too speculative. It might be ok in the discussion. "Overall, these trade-offs suggest that participants prioritized what to observe based on cost; the opponent's betrayal was more costly than losing some control over their own avatar. This cost-driven observation became a natural source of information asymmetry. That is, by shifting their gaze to the opponent, participants gained more information about unexpected competitiveness; otherwise, they learned nothing new. Our results suggest that this difference in information from selective observation eventually biased the later inferences."

We agree that this part may sound speculative, so we removed it from the Result section.

4. RNN: I am not an expert on RNNs and therefore I cannot comment on the details. But I definitely do not like the word "causal" in the following sentence: "To verify whether gaze target shifts causally influence inferential bias, we developed a task optimized recurrent neural network (RNN)"

We changed the phrasing from suggesting a causal relationship to indicating an implied relationship as follows:

**[Line 806-807]** To explore the relationship between gaze target shifts and inferential bias, we developed a task-optimized recurrent neural network (RNN) using the following steps (Fig 5A).

5. Fig 1 on POMDP. I know more about POMDPs than on RNNs. I generally like the background given in Fig 1 but the authors did not use a POMDP to simulate participants' data.

a. In Fig 1, it is slightly confusing that capital "I" is used both for others' Intentions and self's Inference.

We apologize for the confusion. The symbol for others’ intention has been replaced with the symbol “θ” in the revised manuscript.

b. It is also slightly confusing that the observations of the other are included. Wouldn't that be seeing the others' eye gaze in this setup? But here the opponent clearly has no eye gaze.

That is right. In our current study, we did not manipulate or explicitly model the observation of the opponent. Our focus was strictly on the competition between the other's action and the self's action. We included Figure 1 to illustrate all potential variables that could compete for observational resources. We agree that exploring the role of these other variables would be a great direction for future research, and we had elaborated on this idea in the original Discussion section as

**[Line 490-492]** For example, a study demonstrated that manipulating the gaze direction of computer agents altered observers' inferences about animacy

c. I like that colour code seems to correspond to the task layout. But then "green" should be for the self's actions.

We revised the color scheme for better consistency. Thank you for pointing this out.

6. Task design & additional task changes due to F:

a. Did participants know this and did they see this on a trial-by-trial basis? "To prevent participant demotivation, if the opponent caught the prey before the player, the participant received a small portion of the score they would have earned by catching it themselves. Specifically, the original score was multiplied by the opponent's F value and 0.5."

Yes, participants were verbally informed of the related procedure before the experiment. Furthermore, they were able to observe the score obtained for the current trial immediately after the pursuit stage, even if they failed to capture the prey (see Fig. 2A).

b. Was the following really necessary? "To encourage accurate predictions of the opponent's F value during the item decision period, the player's speed in the pursuit period was slightly adjusted based on decision accuracy. Specifically, when the opponent's behavior was predictable (F = 1 or F = 0) and the choice was correct, the player's speed increased to match the prey's maximum speed. Conversely, if the choice was incorrect, the speed difference from the prey could increase up to twice the original gap. When the opponent's behavior was ambiguous (F = 0.5), the player's speed remained unchanged regardless of the participant's choice.

This design was necessary to prevent participants from adopting a strategy of always choosing the hinder item. During game development, we observed that a few testers with exceptional motor skills could capture the prey alone, even without help from the friendly opponent. For these participants, the complacent choice would be to simply bypass the inference task and select 'hinder,' and then rely solely on their own skill. To discourage this undesirable behavior, we provided a small, additional reward for making the correct inference.

7. A potential strategy: As far as I understand, the authors assume that the participants maximize their gains by trying to catch the prey during the pursuit phase. But participants might also try to explicitly check what the opponent does when they move away from the prey. Could such behavior be seen?

This is an interesting idea. However, such behavior was only rarely observed in our experimental setting, likely because it is not generally beneficial to the player. The opponent is fundamentally faster than the player’s avatar (110% of the player’s speed) and therefore will typically capture the prey first, unless the F value is exactly 1—an extreme and highly specific case. That said, we agree that systematically examining individual differences in strategy across subjects and situations would be an interesting direction for future work.

8. "The magnitude of F change was randomly set between +/-0.35 and 0.45." Should this be "between -0.45 and 0.45"?

We apologize for the lack of clarity. Our intended meaning was that ΔF values ranging from -0.35 to -0.45 were employed for the betrayal condition, and +0.35 to +0.45 were employed for the unexpected help condition. We have clarified this specific detail in the text as:

**[Line 643-645]** The ΔF values were randomly set from −0.35 to −0.45 in the betrayal condition and from +0.35 to +0.45 in the unexpected-help condition.

9. Typo in line 739: "F > 0.76" should be "F > 0.67"

We appreciate the reviewer for identifying those errors. The relevant parts have been corrected in the revised manuscript.
