# Supplementary material for: Selective observation following betrayal shapes the social inference landscape
Source: PLoS Comput Biol. 2026 Apr 24;22(4):e1014200. doi: 10.1371/journal.pcbi.1014200 (PMC13193612; doi:10.1371/journal.pcbi.1014200)
Supplement: Attachment — Submitted filename: Response to Reviewers.docx [file pcbi.1014200.s016.docx]

Reviewer #1: The authors have addressed all of my concerns.

We thank the reviewer for the comment and for reviewing the revised manuscript.

Reviewer #2: Thank you for submitting the revised version of the manuscript. I have reviewed the authors' responses to my previous comments and the corresponding modifications made to the paper. I am pleased to confirm that all the concerns and questions I raised have been adequately addressed and resolved. The revisions have significantly enhanced the clarity, accuracy, and overall quality of the work. Based on this, I recommend acceptance of the manuscript in its current form.

We thank the reviewer for all the helpful comments.

Reviewer #3: The authors have substantially improved the manuscript, in particular, strengthened the presented evidence with further analyses and improved the clarity and transparency of their methods. However, the following two points should still be addressed before publication.

We thank the reviewer for the constructive comments. We have addressed the remaining points and revised the manuscript accordingly.

1) The novel control analyses for psychological factors are helpful to rule out several alternative explanations. However, as it stands, the analysis regarding loss aversion is not quite clear to me and should be further elaborated: How exactly does this capture loss aversion? Isn't reward known only _after_ a trial and hence cannot be a modulating factor (i.e., I cannot fear what I don't know)? Also, Fig 2F shows that reward is a monotonic function of given F, so shouldn't perceived F also be (at least weakly) positively linked to reward?

We thank the reviewer for pointing out that our description of this analysis was not sufficiently clear. In S2A Fig and S2B Fig, perceived F refers to the probability of choosing the boost item on the “*subsequent”* trial, whereas reward corresponds to the outcome obtained on the “*previous”* trial. Thus, this analysis examines whether the reward received on the previous trial influences participants' subsequent choice behavior. By contrast, Fig 2F illustrates the relationship between the given F value and the reward obtained on the *same* trial, which reflects the reward structure of the task rather than participants' subsequent choices.

To clarify this point, we have revised the text and figure legends as follows:

**[Line 186 to 190]** However, perceived F values on the next trial did not vary with reward magnitude on the previous trial (S2A Fig; slope, t_47_ = 1.237, p = 0.222). The same result held when we compared the probability of choosing the boost item on the next trial following smaller versus larger rewards on the previous trial (S2B Fig; lower- vs higher-reward, t_47_ = -0.802, p = 0.427).

**[Line 698 to 705]** To assess the sensitivity to loss, we also normalized trial-wise rewards to a 0–1 range for each participant and divided them into 20 equal bins, then measured perceived F (i.e., the probability of choosing the boost item on the next trial) within each bin (S2A Fig, left panel). For each participant, we quantified changes in perceived F (i.e., probability of choosing 'boost' item on the next trial) as a function of reward magnitude on the previous trial using the slope of a linear regression (S2A Fig, right panel). We then performed a median split on the previous trial's reward magnitude and compared slopes between lower-reward and higher-reward trials (S2B Fig).

**[Line 1176 to 1182]** S2 Fig. Reward magnitude had little effect on perceived F. (A) Perceived F (i.e., the probability of choosing the boost item on the subsequent trial) as a function of normalized reward magnitude on the previous trial. The slope reflects each participant's sensitivity to the reward from the previous trial. (B) Relationship between the slopes for low-reward and high-reward trials across participants. (C) Mean perceived F following betrayal and unexpected-help conditions within the just-noticeable-difference range. Error bars and shaded regions represent ±1 SEM; dots indicate individual participants.

2) It appears that the sample size from study 1 and various test statistics have been altered, but not highlighted in the manuscript. Can the authors provide a rationale why these additional subjects were added, and state if any findings and conclusions from the paper were affected by including them? One response to reviewer mentions replications, but the subjects seem to have been added to study 1 rather than as separate replication sample. Relatedly, the different samples should be clearly separated throughout the manuscript (e.g., the Results section starts by reporting the total N, but then goes on to report results based only on the subjects from study 1).

In the previous review round, another reviewer (reviewer 1) noted that the current study was not preregistered. Following this comment, the editor recommended that we include additional data or analyses to demonstrate the robustness and replicability of our findings. In response to this recommendation, we recruited an additional eight participants to examine whether the same pattern of results would also be observed in additional participants.

We found that these participants showed a comparable pattern to the original sample. Therefore, we incorporated them into the original sample and updated the corresponding statistical values. Importantly, the inclusion of these additional participants did not change the results or conclusions drawn from the statistics reported in the initial manuscript.

We now clarify the rationale for including these additional participants and report their results separately in S1B Fig and S7B Fig as follows:

**[Line 164 to 165]** Eight participants in the present sample were recruited in a separate session to assess the robustness of this effect, and showed a comparable pattern of results (S1B Fig).

**[Line 311 to 314]** As with S1B Fig, eight participants in the present sample were recruited in a separate session and showed the same gaze-shift pattern toward the opponent in betrayal trials but not in unexpected-help trials (S7B Fig).

Reviewer #4: The authors have answered all my questions. My suggestion for minor revisions is that they should also include the explanaitons for the design choices and the methodological aspects into

We thank the reviewer for constructive suggestions. We have incorporated the explanations for the design choices into the Methods section as follows:

**[Line 558 to 561]** The inference phase was introduced to allow participants to form an expectation about the opponent's role before making a decision. Separating this phase from the pursuit phase also prevents the inference process from being confounded by factors such as motor planning and execution.

**[Line 658 to 661]** The block structure was used to allow participants to accumulate interaction history under a consistent violation pattern. To focus on expectation violations rather than gradual learning of the opponent's role, the F value was randomly assigned on each trial.
